# Snapback Primer Mediated Clamping PCR for Detection of *EGFR* and *KRAS* Mutations in NSCLC Patients by High Resolution Melting Analysis

**DOI:** 10.1155/2014/407537

**Published:** 2014-05-04

**Authors:** Haiyan Sun, Yang Yang, Lixin Yang, Bo Su, Gening Jiang, Ke Fei, Daru Lu

**Affiliations:** ^1^State Key Laboratory of Genetic Engineering, Fudan-VARI Genetic Epidemiology Center and MOE Key Laboratory of Contemporary Anthropology, School of Life Sciences, Fudan University, 220 Handan Road, Shanghai 200433, China; ^2^Department of Thoracic Surgery, Shanghai Pulmonary Hospital, Tongji University School of Medicine, 507 Zheng Min Road, Shanghai 200433, China; ^3^Department of Thoracic and Cardiovascular Surgery, Second Military Medical University, Changhai Hospital, Shanghai 200433, China; ^4^Department of Central Laboratory, Shanghai Pulmonary Hospital, Tongji University School of Medicine, 507 Zheng Min Road, Shanghai 200433, China

## Abstract

Assays for detecting somatic mutations are requested with higher sensitivity and more convenience. Here, we describe snapback primer mediated allele clamping enrichment polymerase chain reaction (SPACE-PCR), a novel form of PCR that amplifies minority alleles selectively from mixtures. We replaced regular PCR with SPACE-PCR before sequencing or genotyping assays to improve mutation detection sensitivity by up to 100-fold in detecting *EGFR* and *KRAS* somatic mutations. Combined SPACE-PCR with analysis of snapback primer by high resolution melting (SPACE-HRM), the high sensitive system that enables a closed-tube detection of mutations after isolating mutants has been established, as low as 1/10^5^–1/1000 mutant samples can be diagnosed. And finally, in a double-blind experiment of 150 cases of non-small-cell lung cancer (NSCLC) patients, compared with direct DNA sequencing and ADX-*EGFR/KRAS* mutation detection kit, up to 25% of the PCR-direct sequencing negative cases turned out to be positive in SPACE-HRM mutation tests; the specificity is 100%. Results demonstrated that the SPACE-HRM system we set up is a high sensitive assay that can be used for *EGFR* and *KRAS* allele enrichment and reliable detection. We anticipate that the method will be employed in multiple applications in the clinic, including diagnosis, scanner recurrence monitoring, and treatment management.

## 1. Introduction

Somatic mutations in the gene of epidermal growth factor receptor (*EGFR*) and* KRAS* are associated with sensitivity and resistance to the kinase inhibitors in targeted therapy. Detection of* EGFR* and* KRAS* mutations is now a necessary procedure for treatment of non-small-cell lung cancer (NSCLC) before using* EGFR* tyrosine kinase inhibitors (*EGFR*-TKI) [1–4]. For the heterogeneity in tumor tissues and dilution of wild type, the ratio of mutant to wild type DNA could be less than 10%. The methods must be sensitive enough to detect such low-frequency mutant samples.

**Figure 1:**
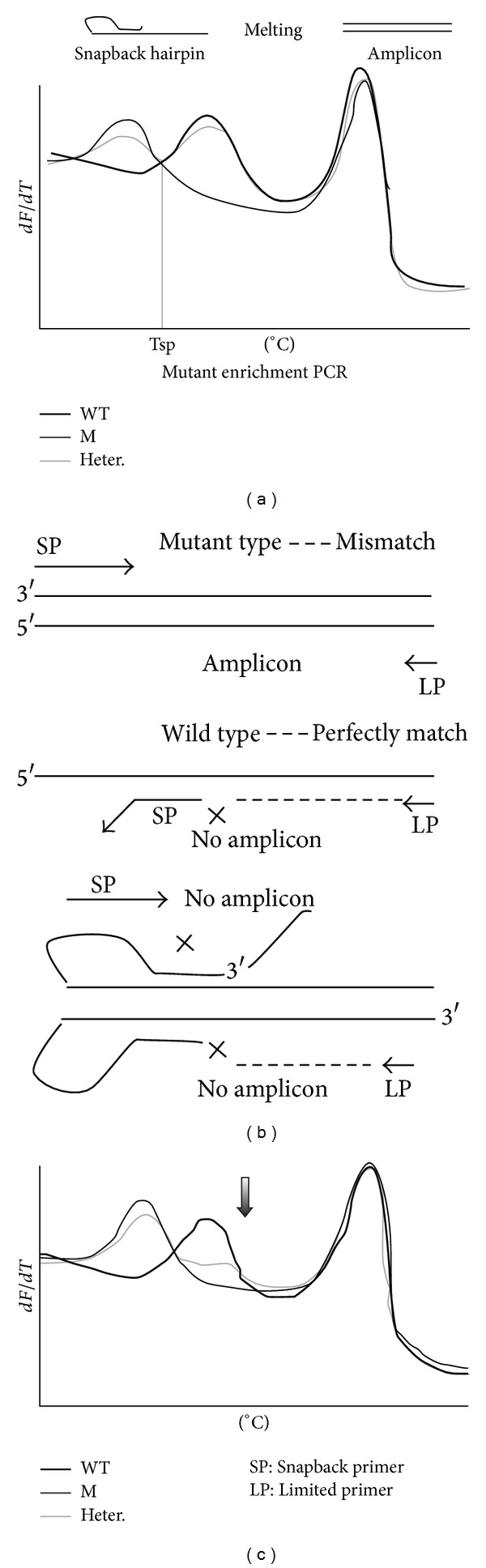
Snapback primer mediated clamping PCR and HRM analysis. We designed probe's sequence that matches wild type to make sure that the melting temperature of wild hairpin stem structure will be higher than mutant type. (a) HRM analysis by snapback primer genotyping after normal PCR. Tsp value is the middle melting temperature of wild and mutant snapback hairpin, which is the temperature chosen for extending in SPACE-PCR cycles. (b) The principle of SPACE-PCR. When extended in Tsp (°C), mutant can be enriched. (c) HRM analysis with snapback primer after SPACE-PCR (SPACE-HRM). After SPACE-PCR, both the melting curves of wild snapback hairpin and amplicon are reduced. Heterozygous samples intend to be pure mutant.

Although direct DNA sequencing is often considered as a gold standard for the identification of mutations, it remains laborious and time consuming. Moreover, it might suffer from a lack of sensitivity about 10%–20% and robustness for the determination of mutations [5]. Alternative methods have therefore been developed to detect these common mutations based on restriction enzyme analysis, allele specific amplification, digital PCR, fluorogenic hybridization probes, and high resolution melting (HRM) [6–9]. HRM analysis is a post-PCR technique to identify genetic variation in nucleic acid sequences. It has been used to screen large numbers of potential variant sequences and genotype mutations in* EGFR* and* KRAS* detections [3, 10, 11]. As a one-step precursor to other more laborious techniques such as sequencing, it has incomparable superiority in clinical promotion. In 2008, Zhou et al. developed snapback primer into genotyping analysis by HRM without extra cost of special covalent modifications. After several thermal cycles of amplification, dependent on the amplicon sequence of snapback primer, a complementary probe portion of primer tail snaps back on its extension products so that an intramolecular hairpin is formed after the asymmetric PCR; then the melting curves of snapback hairpin were analyzed based on the difference of 3–6°C between wild and mutant genotyping [12]. In this study, we developed a snapback primer mediated allele clamping enrichment polymerase chain reaction (SPACE-PCR) for mutant-enrich amplification and HRM analysis in detecting* EGFR* and* KRAS* mutations. When in the cycling of amplification, the extending temperature is set between the wild and mutant melting temperature of hairpin stem structure to inhibit the amplification of wild template, while the mutant comes to be enriched successfully. Then, cooperated with analysis of snapback primer by high resolution melting (SPACE-HRM), mutant peaks must be more obvious, while wild peaks are weakened (Figure 1).

We developed SPACE-HRM assays for* EGFR* and* KRAS* common mutations that are currently used for drug sensitivity and resistance diagnoses. The enrichment efficiency of the SPACE-PCR was tested with sequencing and HRM analysis using a dilution series of mutant DNA into wild DNA. Furthermore, we showed that false-negative results analyzed by method of direct DNA sequencing or Scorpion-ARMS could be identified by SPACE-HRM in a test of 150 cases of NSCLC patient samples.

## 2. Materials and Methods

### 2.1. DNA Samples

Tumor genomic DNA of NSCLC patients were obtained from our previous study [11] and Tongji University affiliated Shanghai Pulmonary Hospital. We extracted paraffin tissues and fresh tissues with QIAamp DNA FFPE Tissue Kit and QIAamp DNA Mini Kit (QIAGEN, Germany) according to the instructions of the manufacturer. DNA samples were diluted in ddH_2_O and then measured by the absorbance at 260 nm (NanoDrop 2000; Thermo Fisher Scientific). The final concentration was 20 ng/mL.

### 2.2. Primers

Snapback primer consists of unlabeled probe attached to the 5′-end of PCR primers without any modifications. A 2-bp mismatch at the 5′-end of the snapback primer may also be included to prevent 3′-end extension of the minor hairpin that may form from the full-length single strand that includes the limited primer. We designed primers to detect L858R, T790M, 19 deletions in* EGFR* and exon 2, codons 12 and 13 mutations in* KRAS*. Sequences of primers are shown in Supplementary Table 1 (see Supplementary Table 1 in Supplementary Material available online at http://dx.doi.org/10.1155/2014/407537).

### 2.3. PCR and Melting Curve Acquisition

10 *μ*L PCR reaction volumes containing 50 mmol/L Tris (pH 8.3), 500 mg/L bovine serum albumin, 3.5 mmol/L MgCl2, 200 *μ*mol/L of each deoxynucleotide triphosphate (dNTP), 0.4 units TaKaRa HS polymerase (TaKaRa, Code: DR010), DNA samples (20 ng tissue gDNA), 1 × LC Green Plus (Idaho Technology), 0.4 *μ*mol/L of the snapback primer, and 0.04 *μ*mol/L of the limiting primer.

The amplification of all the mutations detected in* EGFR* and* KRAS* was adjusted to the same program in Light Cycler 480 II (LC480 II, Roche) for 55 cycles with denaturation at 95°C (15 s hold), annealing at 65°C (10 s hold), and a 2°C/s ramp to the extension temperature of 75°C (15 s hold) for normal PCR and 69°C (15 s hold) for SPACE-PCR.

After PCR, the products were denatured at 95°C (60 s hold), cooled to 55°C (60 s hold), and melted at 0.02°C/s with continuous acquisition of fluorescence until 95°C. After HRM analysis, the reaction volumes are delivered to DNA sequencing.

### 2.4. Melting Curves Analysis

The melting of both intramolecular snapback hairpins and intermolecular amplicon duplexes is observed as peaks on negative first-derivative plots of fluorescence with respect to temperature by analysis method “Tm calling” that instrument LC480 II provides.

## 3. Results

### 3.1. The Establishment of SPACE-HRM

**Figure 2:**
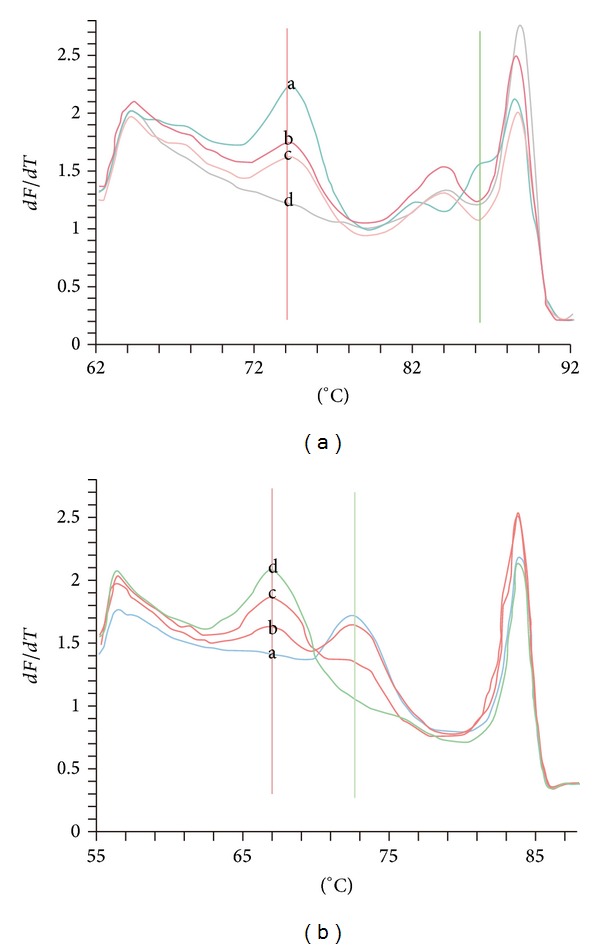
A schematic of genotyping by snapback primer HRM after normal PCR in detecting* EGFR* E746-A750 del (Figure 1(a)) and* KRAS* exon 2, codon 13(1-G>A). In detection of* EGFR* E746-A750 del (15 bp), probe (22 bp) cannot cover the mutant site, so there is no peak of intramolecular snapback hairpins for mutant-type samples, while the peak value of wild type is 74.5°C. Besides this, difference also exists in peaks of intermolecular duplexes. When there is an obviously small peak 2°C below the main peaks of intermolecular duplexes, it means this sample just consists of wild sequence (peak a); then if 4°C-below, mutant exists (peaks b, c, and d). Mixed samples (peak b, 10% mutation, and peak c, 20% mutation) showed that the more mutant template the lower probe peak. In detection of* KRAS*, as for point mutation detection, wild-type (peak a) probe peak value is 74°C and about 5°C below is the mutation (peak d). Mixed samples (peak c, 50% mutation, and peak b, 20% mutation) showed that the higher the percentage, the higher the peak standing for.

A characteristic of genotyping deletion and point mutant by snapback primer HRM after normal PCR is shown in Figure 2. For a large fragment of deletion (E746-A750 del (15 bp del)), probes we designed could not snap back with mutant to form a hairpin stem, with showing full-length amplicon melting only. For point mutations (*KRAS* exon 2, codon 13 (1-G>A)), two different melting peaks of snapback hairpin were mutant and wild sample, respectively.

**Figure 3:**
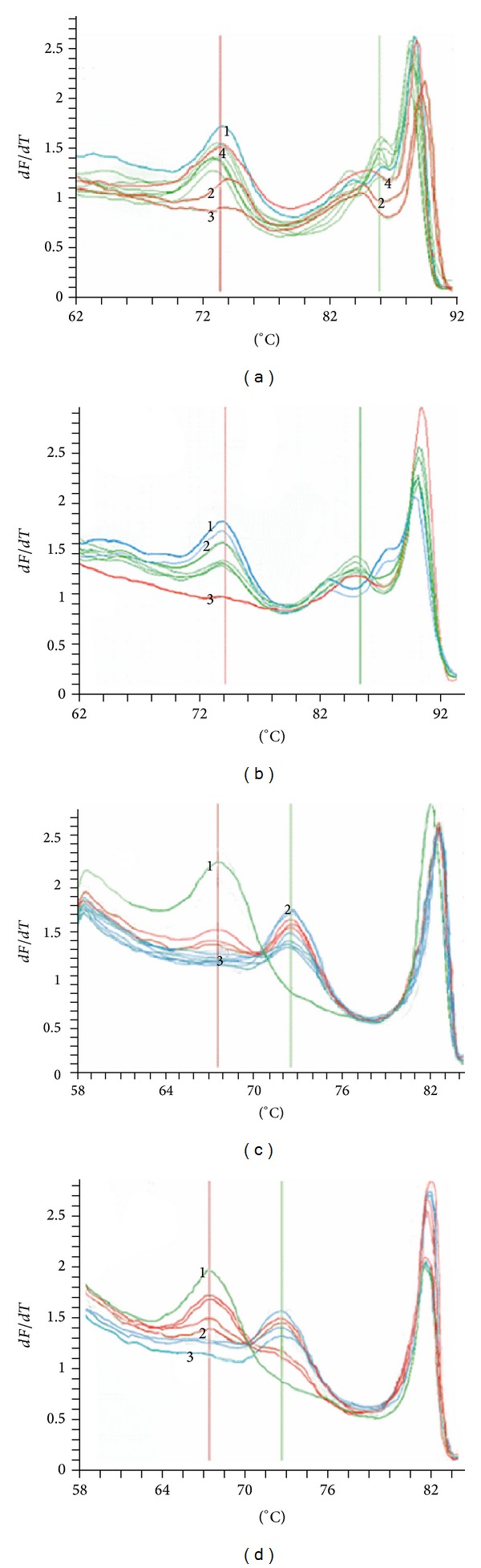
Sensitivity of snapback primer HRM after normal PCR and SPACE-PCR. Mutations accounted for 100%, 1/10, 1/100, 5/1000, 1/1000, 1/10^4^, and 1/10^5^ and wild samples were tested. (a) The sensitivity-test of* EGFR* E746-A750 del by snapback primer HRM after normal PCR. Lines 1 and 4 are pure wild and mutant, while lines 2 and 4 are 1/100 and 1/10 mutant sample. Here, the 1% detection sensitivity is shown. (b) The sensitivity-test of* EGFR* E746-A750 del by SPACE-HRM. Lines 1, 2, and 3 stand for wild, 1/10^5^ mutant, and 100% mutant sample, respectively. Now, sensitivity was improved to 1/10^4^. (c) The sensitivity-test of* KARS* mutation by snapback primer HRM after normal PCR. Lines 1, 2, and 3 stand for 100% mutant, wild, and 1/100 mutant samples; more than 1% of mutant sample can be detected. (d) The sensitivity-test of* KARS* mutation by SPACE-HRM. Lines 1, 2, and 3 stand for 100% mutant, 1/1000 mutant, and wild samples; more than 1% of mutant sample can be detected. Compared with (a) and (b), SPACE-PCR improves sensitivity of normal snapback primer HRM to 1/1000.

Then, the sensitivity of SPACE-HRM was compared with snapback primer HRM after normal PCR in Figure 3. Mutations accounted for 1, 1/10, 1/100, 5/1000, 1/1000, 1/10^4^, and 1/10^5^ and wild samples were tested. Before enrichment, 1/10 mutant sample was the baseline. For deletions, it seems to be enriched more easily by SPACE-HRM. 1/10^5^ mutant samples could be detected, while 1-bp mutation was improved to be 1/1000.

**Figure 4:**
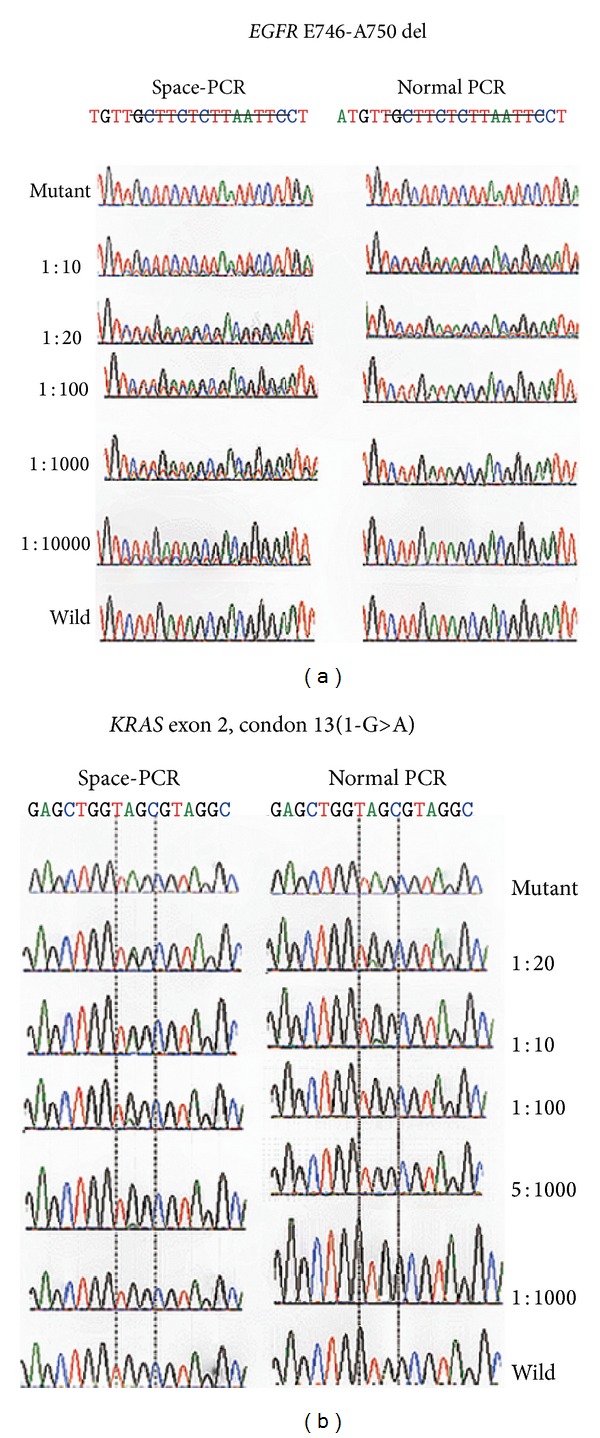
Enrichment efficiency of sequencing with SPACE-PCR. Mutant and wild samples were mixed into different ratio from 1/20 to 1/10000. After SPACE-PCR and normal PCR, detect* EGFR* E746-A750 del and* KRAS* exon 2, codon 13 (1-G>A) by DNA sequencing. In the figures of* EGFR* 19 del, after SPACE-PCR, 1/10000 mutant is detected, while the normal PCR is just 1/10. To point mutations, as* KRAS* detected here, 1/10 sensitivity is improved to be 5/1000 by SPACE-PCR.

Finally, to make sure of the enrichment efficiency, the enrichment results of SPACE-PCR were verified by DNA sequencing. Sequence diagrams of SPACE-PCR and normal PCR were compared in Figure 4. The sensitivity of direct sequencing was about 1/10. After mutant enrichment by SPACE-PCR, 1/10^4^ deletion mutant-type and 1/200 point mutation have been analyzed.

### 3.2. Double Blind Testing of 150 Tissue Samples Compared with Sequencing and ADX-EGFR/KARS Mutation Detection Kit

We extracted DNA, coded number 1-number 150, from 50-paraffin tissue and 100 fresh tissue samples. Then, SPACE-HRM, direct DNA sequencing, and Scorpion-ARMS simultaneously diagnosed these samples with ADX-*EGFR*/*KARS* mutation detection kit (Amoy Diagnostics Co. Ltd., Xiamen, China).

To compare the methods between SPACE-HRM, direct DNA sequencing, and Scorpion-ARMS with ADX-*EGFR*/*KARS* mutation detection kit (Amoy Diagnostics Co. Ltd., Xiamen, China), DNA from 50-paraffin tissue and 100 fresh tissue samples coded from number 1 to number 150 were extracted and used to test simultaneously.

48 patients (49/150; 32%) were found to harbor activating* EGFR* and* KRAS* mutations using PCR-direct sequencing method, with a majority of patients (39/49; 80%) carrying exon 19 deletion or L858R point mutations. 101 cases negative for* EGFR* and* KRAS* mutations from PCR-direct sequencing were further analyzed using Scorpion-ARMS and SPACE-HRM technology. Up to 25% of the PCR-direct sequencing negative cases turned out to be positive in SPACE-HRM mutation tests, and this proportion is 20% by the Scorpion-ARMS. The amount of patients with different mutation by the three assays was in Table 1. Mutant details were listed in Supplementary Tables 2 and 3.

## 4. Discussions

Currently, DNA sequencing is also the “golden standard” of genotyping technologies, and Scorpion-ARMS is thought to be the most efficient method in detecting* EGFR* and* KRAS* mutations, which can be used to analyze mutations higher than 1%, and FDA US has accredited it in detection of* EGFR* and* KRAS* mutations. The specificity of SPACE-HRM is tested compared with these two standard methods. Results were convincing; the SPACE-HRM detection system might be a good choice for one-step mutation assay for its highest sensitivity.

A key limitation of PCR-based methods is the inability to selectively amplify low levels of mutations in a wild-type background. As a result, downstream assays are limited in their ability to identify subtle genetic changes that can have a profound impact in clinical decision-making and outcome. For the purpose of obtaining high sensitivity, the mutant-enrich PCR combined with high sensitive post-PCR analysis methods must be necessary for PCR-based assay. SPACE-PCR that enables exclusive amplification and isolation of the mutants would transform the capabilities of PCR-based genetic testing. It is operated in an easy way by changing extending temperature of thermal cycles. For mutation of large-fragment insertions and deletions, when probes cannot hybridize to the template, we can choose whatever temperature under the wild melting temperature of hairpin. For point mutations and small fragment mutants, temperature between mutant-type and wild-type hybridizing temperature is necessary. The difference is about 5°C, certainly closer to the wild and more efficient to mutant enrichment. But we often select the middle 2-3°C to make sure of the mutant-enrichment as well as the low-frequency mutant samples be amplification successfully. Compared with another enrichment method-COLD PCR, the temperature used for enrichment is easier to choose, which means the experiment's results can be more stable with being little affected by PCR instruments. Normal PCR thermal cycling instruments can well perform the method for mutant-enrich PCR. We have made a test in common PCR instruments with the same protocol described above, and the same results were obtained.

SPACE-PCR can be combined with any post-PCR analysis method, if sensitive enough, like HRM, digital PCR, and so on [13]. Ultrahigh sensitive assay will be developed. Here, we chose the snapback primer HRM and formed a simple and one-step platform for clinical use.

Nowadays, many high sensitive technologies were developed to detect circulating cell-free DNA (cfDNA) in plasma or serum [11, 14–16], which is useful for numerous diagnostic applications and would avoid the need for tumor tissue biopsies. Since large difference of cfDNA concentration and integrity exists in different samples, methods with cluster analysis that need a uniform concentration of starting template seem to be not ideal, such as PNA/LNA-mediated real-time PCR clamping, TaqMan probe ARMS, and Scorpion-ARMS primers for SNP real-time PCR detection [1, 17–19]. We distinguish wild and mutant by the shape of the melting peaks instead of the amplification Ct values, which means the concentration homogenization of DNA before reaction and the cluster analysis are not needed. A small amount sample of pretest has been done in cfDNA detection, and results were consistent with what we expected. Besides, it can be better for use, when samples are not fresh or are extracted with the different methods. Precisely because of its high sensitive and intuitive way of distinction without comparisons of references, our high sensitive SPACE-HRM system may be a good choice for circulating peripheral blood analysis. The experiment in this area continues to be done.

## 5. Conclusions

We developed a new method for mutant-enrich PCR only with snapback primer (SPACE-PCR), in which the enrichment efficiency is nearly 100-fold. The efficiency is particularly obvious for large fragment mutations. With this method, we can detect lower than 1/10 mutant samples by sequencing. Then, new snapback primer HRM assays with innovative SPACE-PCR technology (SPACE-HRM) were specifically developed to detect somatic* EGFR* and* KRAS* mutations that are currently used for drug sensitivity and resistance diagnoses. By combining the power of the two technologies, a simple, fast, highly sensitive, and high throughput DNA detection system for one-step detection of clinic application was established, with which we can detect 1/1000–1/10000 low-mutant samples. In large fragment mutations, like* EGFR* 19 del (15 bp) we designed in this experiment, detection ratio reached 1/10^5^. Therefore, the SPACE-HRM system is a high sensitive assay that can be used for extremely low-frequency mutant samples.

## Supplementary Material

Supplementary information provides DNA sequences and detailed double-blind results associated with the experiment.

## Figures and Tables

**Table 1:** The amount of patients with different mutations detected by SPACE-HRM, sequencing, scorpion ARMS.

Amount	SPACE-HRM	Sequencing	Scorpion-ARMS
*EGFR* 19 del	33	21	29
L858R	27	20	26
T790M	7	5	7
*KRAS *	15	10	13

Total	83 (11 double mutant)	56 (8 double mutant)	75 (11 double mutant)
