# Supplementary material for: Snapback Primer Mediated Clamping PCR for Detection of EGFR and KRAS Mutations in NSCLC Patients by High Resolution Melting Analysis
Source: Biomed Res Int. 2014 May 4;2014:407537. doi: 10.1155/2014/407537 (PMC4026848; doi:10.1155/2014/407537)
Supplement: Supplementary file 1 — Supplementary information provides DNA sequences and detailed double-blind results associated with the experiment. [file 407537.f1.doc]

Supplementary information

Table 1: Primer sequences used in this experiment

| Mutation Site | Primer Name | Primer Sequence(5`-3`) |
| --- | --- | --- |
| *EGFR*  exon19 del | Snapback primer 1 | aaCGGAGATGTTGCTTCTCTTAATTCCTTGATAGTCTGTC  ATAGGGACTCTGGAT |
|  | Limited primer 1 | AAGGGAAAGACATAGAAAGTGAACATTTAGGA |
| L858R | Snapback primer 2 | taCACAGATTTTGGACTGGTCAAACTGCGCCACCTCCTTACTTTGCC |
|  | Limited primer 2 | CCATGATGATCTGTCCCTCACAGCAG |
| T790M | Snapback primer 3 | aaAGCTCATCACGCAGCTCAGCCAGTTGAGCAGGTACTGGGAG |
|  | Limited primer 3 | GCGTCTTCACCTGGAAGGGGTCCA |
| *KRAS* Exon2  Codon 12/13 | Snapback primer 4 | atTGGAGCTGGTGGCGTAGTTGGATCATATTCGTCCACA |
|  | Limited primer 4 | TACTGGTGGAGTATTTGATAGTGTATTAACCTT |

| Table 2:The sample NO. of different mutations detected by SPACE-HRM, sequencing, scorpion ARMS. | | | | | | |
| --- | --- | --- | --- | --- | --- | --- |
| Mutation NO. | SPACE-HRM | | Sequencing | | scorpion-ARMS | |
| *EGFR* 19 del | 4,8,12,18,26,27,33*,34*,38, 42*,  45,47,61,63*,69,73*,76,77,79,80,  83,85,88,94,97,101,105,109**,126,131,136**,137**,147 | | 4,8,12,18,26,27,38,45,47,61,69,77,  79,80,85,88,94,105,126,131,147 | | 4,8,12,18,26,27,33*,34*,38,42*,45,47,61,63*,69,73*,76,77,79,80,83,  85,88,94,97,101,105,126,131,147 | |
| L858R | 2,5,7,12,17,33,37,40,45,48,50,52*,  57*,62,68,75,80*,82,90*,96,105, 113*,115,122,137, 144,147** | | 2,7,12,17,33,37,40,45,48,50,62,68,  75, 82, 96,105, 115,122,137,  144 | | 2,5,7,12,17,33,37,40,45,48,50,52*,  57*,62,68,75,80*,82,90,96,105,  113*,115,122,137, 144 | |
| T790M | 27,33,69*,85,109,115*,144 | | 27,33,85,109,144 | | 27,33,69*,85,109,115*,144 | |
| *KRAS* | 2*,11,19,23*,31**,39,44,49,53,  54*,72,87**,92,110,135 | | 11,19,39,44,49,72,53,92,110,135 | | 2*,11,19,23*,39,44,49,53,54*72,92,110,135 | |
| *KRAS* |  |  |  |  |  |  |

Samples with‘*’is just detected by SPACE-HRM and scorpion-ARMS, and with ‘**’ , it is just detected only by SPACE-HRM.
